# Vitamin D Receptor Polymorphisms and Their Association with Nrf2-Dependent Gene Expression in Oxidative Stress Resilience in Cognitively Healthy Aging

**DOI:** 10.3390/antiox15080916

**Published:** 2026-07-23

**Authors:** Bárbara Bruna, Nohela Arévalo-Ramírez, Daniela P. Ponce, María Isabel Behrens, Carol D. SanMartín

**Affiliations:** 1 Centro de Investigación Clínica Aplicada (CICA), Hospital Clínico Universidad de Chile, Santiago 8380456, Chile; 2 Centro de Biomedicina, Universidad Mayor, Santiago 8580745, Chile; 3 Departamento de Neurología y Neurocirugía, Facultad de Medicina, Universidad de Chile, Santiago 8380456, Chile; 4 Instituto de Nutrición y Tecnología de los Alimentos (INTA), Universidad de Chile, Santiago 7830490, Chile

**Keywords:** Vitamin D, aging, vitamin D receptor (VDR), redox signaling, VDR polymorphisms, Nrf2

## Abstract

The vitamin D receptor (VDR) exerts pleiotropic effects essential for human health and modulates the transcription of nuclear factor erythroid 2-related factor 2 (Nrf2), a key regulator of the cellular antioxidant defense system. Polymorphisms represent a major source of interindividual variability and may influence susceptibility to oxidative stress. The VDR polymorphisms ApaI (A > C) and TaqI (T > C) have been associated with disease susceptibility; however, their role in oxidative stress responses during aging remains poorly understood. This study evaluated the association between ApaI and TaqI polymorphisms and susceptibility to oxidative stress-induced cell death, as well as the expression of genes within the VDR–Nrf2 signaling axis, in peripheral blood mononuclear cells (PBMCs) from cognitively healthy Chilean older adults. VDR genotyping was performed using real-time PCR (TaqMan SNP Genotyping Assay), PBMC viability following H_2_O_2_ exposure was assessed by flow cytometry, and gene expression levels were determined by quantitative PCR (qPCR). For ApaI, homozygous carriers of the A allele showed greater PBMC viability following H_2_O_2_ exposure (*p* < 0.0068) and higher mRNA expression levels of Nrf2 (*p* = 0.0176) and its downstream genes HMOX1 (*p* = 0.0070) and GST (*p* = 0.0047) compared with carriers of the C allele (AC or CC). For TaqI, homozygous carriers of the protective C allele exhibited greater PBMC viability (*p* < 0.0465) and higher Nrf2 (*p* = 0.0324), HMOX1 (*p* = 0.0206), and GST (*p* = 0.0029) expression compared with carriers of the risk T allele (TC or TT). These findings provide novel evidence that VDR polymorphisms may modulate antioxidant responses through regulation of Nrf2-dependent gene expression, suggesting a genetic determinant of oxidative stress resilience in cognitively healthy older adults.

## 1. Introduction

Vitamin D is a multipurpose secosteroid hormone classically associated with Ca^2+^ homeostasis, bone formation and heath. However, in recent years, other functions and roles have been verified, and currently, it is accepted that vitamin D has pleiotropic effects [1].

The epidermis is the major site for sunlight-mediated vitamin D synthesis, while that obtained by intake corresponds to 10–20% [2,3]. Its biosynthesis starts in the skin, where exposure to UVB radiation synthesizes cholecalciferol from 7-dehydrocholesterol in a nonenzymatic way. After two sequential enzymatic reactions, cholecalciferol is converted to the biologically active 1,25 dihydroxyvitamin D (vitamin D) [3]. The genomic effect of vitamin D is mediated through its interaction with a high-affinity nuclear vitamin D receptor (VDR), a member of the nuclear receptor superfamily of ligand-activated transcription factors. Vitamin D bound to VDR interacts with the retinoid X receptor (RXR); the VDR-RXR heterodimer then recruits coregulatory protein complexes and binds to vitamin D response elements (VDREs) of DNA sequences of vitamin D-regulated genes [4]. Evaluation of genome-wide gene expression influenced by vitamin D supplementation revealed a change in 3% of the genome [5,6]. Some of these genes are transcription factors, as well as BCL6 and Nrf2, which promote the expression of VDR secondary genes. The differentially expressed genes were significantly enriched in immune-related functions, transcriptional regulation, cell cycle activity, DNA replication and stress response [6,7].

Vitamin D levels decline with age, with the elderly being the main group affected [8,9], due to a decrease in the epidermal concentrations of the vitamin D precursor 7-dehydrocholesterol [10]. Low serum concentrations of vitamin D (lower than 30 ng/mL [2]) have been related to aging-related diseases such as osteoporosis, type 2 diabetes mellitus [11,12], Parkinson [13,14], mild cognitive impairment (MCI) and Alzheimer’s disease (AD) [15,16] and increased risk of all-cause mortality. For this reason, vitamin D has been considered one of the modifiable factors that can contribute to better management of aging-related diseases [17,18].

Single-nucleotide polymorphisms (SNPs) are the most common type of genetic variation and are responsible for the genetic diversity observed among individuals. Coding SNPs can lead to changes in the amino acid sequence of the resulting protein affecting its structure or function. Meanwhile, non-coding SNPs can influence gene expression or other molecular processes. Therefore, SNPs can have different effects on an individual’s phenotype and disease susceptibility and are associated with an increased or decreased risk of certain diseases or conditions [19,20]. The impact of SNPs in the return of homeostasis and the wide variation in the severity and age at which individuals are afflicted is still unknown.

A great number of SNPs have been described in the human VDR gene [21,22], but only a few of them have been related to a phenotypic effect such as an altered level of gene expression. The most studied are four SNPs, named according to the restriction enzyme that allowed their identification: Fok I (rs2228570), BsmI (rs1544410), ApaI (rs7975232) and TaqI (rs731236). These polymorphisms have been associated with diseases such as cancer, osteoporosis, tuberculosis, diabetes mellitus and AD [23,24,25].

TaqI (T > C, exon 9) and ApaI (A > C, intron 8) are located near the 3′ untranslated region (3′UTR). Their effect on VDR function remains unclear, but they may influence mRNA stability, transport, and localization. Meanwhile, FokI (T > C, exon 2) shifts the translation start site, resulting in the loss of three amino acids at the VDR protein’s N-terminal end. Due to the VDR response to vitamin D levels, some studies have evaluated the differential response to vitamin D in relation to ApaI and TaqI polymorphisms. The results are controversial, but there seems to be a trend of the presence of the A allele of ApaI and C allele of Taq I displaying a better response in bone mineral density, a lower frailty index and survival to extreme old age [26,27,28]. We [29] and other groups [30,31,32] have reported that these alleles are associated with a lower risk of presenting amnestic MCI, a precursor stage of AD, suggesting that it might be a protective factor against AD development. Additionally, we evaluated the effect of these VDR polymorphisms on the expression of VDR and its target genes that are involved in AD pathogenesis: glycoprotein P (P-gp) and the low-density lipoprotein receptor-related protein-1 (LRP1), both of which mediate beta amyloid (Aβ) clearance, a peptide involved in AD pathogenesis, through the blood–brain barrier. We found that carriers of the AA genotype of ApaI had higher levels of mRNA expression of VDR, P-gp and LRP1 than carriers of one or two copies of the C allele (AC/CC). However, we found no differences in mRNA expression of VDR target genes concerning the TaqI polymorphism [29]. These findings suggested that the presence of the protective alleles could be associated with better Aβ clearance from the brain to the blood, thus explaining the association between VDR polymorphisms and MCI and AD.

Vitamin D has been increasingly recognized for its potential role in modulating antioxidant pathways [33,34] by transcriptional regulation of the nuclear factor erythroid 2-related factor 2 (Nrf2). Nrf2 is a central component of the cellular machinery underlying biological resilience, which refers to the ability of cells to rapidly sense stress and mount appropriate adaptive responses, with the efficiency of these detection and response pathways ultimately determining cell fate [35]. Nrf2 orchestrates the cellular response to oxidative stress by controlling the gene expression of key components of the glutathione and thioredoxin (TRX) antioxidant system, as well as enzymes involved in NADPH regeneration [36]. In basal condition, Nrf2 is sequestered in the cytosol and marked for proteasomal degradation by Keap1, which acts as an adapter for E3-ubiquitin ligase. Electrophiles and signals from reactive oxygen species (ROS) can disrupt the Nrf2/Keap1 interaction, promoting Nrf2 activation and subsequent nuclear translocation [37]. Vitamin D increases Nrf2 expression and promotes its nuclear translocation [38,39]. Moreover, vitamin D has been reported to repress Keap1 transcription through VDR-mediated histone hypermethylation at the Keap1 promoter region, thereby reducing Nrf2 degradation and enhancing its nuclear accumulation at the transcriptional level [40]. Together, these mechanisms position the VDR–Nrf2 pathway as an important regulator of cellular redox adaptation.

However, the influence of polymorphisms of the VDR gene on susceptibility to oxidative stress and on the efficiency of cellular antioxidant responses mediated through the VDR–Nrf2 axis remains insufficiently characterized. Dysregulation of redox homeostasis and reduced cellular resilience are increasingly recognized as early features associated with cognitive decline during aging, highlighting the relevance of this pathway to successful aging. To address this gap, we investigated the impact of the ApaI and TaqI VDR polymorphisms on cellular susceptibility to oxidative stress-induced cell death and expression of Nrf2 and its downstream targets, using peripheral blood mononuclear cells (PBMCs) from cognitively healthy older adults as a peripheral model to explore mechanisms relevant to neurodegenerative vulnerability.

## 2. Materials and Methods

### 2.1. Materials

The EDTA-containing Vacutainer™ was from Becton Dickinson (Macquarie Park, Australia). The High-Capacity cDNA Reverse Transcription Kit and TaqMan^®^ SNP Genotyping Assay were from ThermoFisher (Waltham, MA, USA). Lymphoprep, SepMate and CryoStor solution were from StemCell Technologies (Vancouver, BC, Canada). Ammonium–Chloride–Potassium (ACK) lysis buffer and RPMI 1640 medium were from Gibco. TRIzol^®^ Reagent was from Life Technologies (Carlsbad, CA, USA). Master Mix SsoAdvanced™ Universal SYBR^®^ Green Supermix was from Bio-Rad (Hercules, CA, USA). Anti-Nrf2 antibody and the DNA Genomic PureLink^®^ Kit were from Invitrogen (Carlsbad, CA, USA). Alexa Fluor 488-conjugated was from Jackson InmunoResearch (West Grove, PA, USA). Propidium iodide (PI) and hydrogen peroxide were from Merck (Darmstadt, Germany).

### 2.2. Participants

A total of 30 cognitively healthy older adults of both genders, aged 60 years or older, were recruited in Santiago, Chile, and enrolled in this study (Table 1). All participants provided written informed consent prior to inclusion. The protocol was approved by the Ethics Committee of the Hospital Clínico de la Universidad de Chile (HCUCH). All participants were cognitively evaluated using the Montreal Cognitive Assessment (MoCA) validated in Spanish [41] and the Montreal Cognitive Assessment Memory Index Score (MoCA-MIS). The maximum score for MoCA is 30, with lower scores associated with more important cognitive deterioration and a cutoff for MCI of 21 [41]. The score of the MoCA-MIS ranging from 0 to 15 was calculated by adding the number of words remembered in free delayed recall multiplied by 3, category-cued recall multiplied by 2, and multiple-choice-cued recall multiplied by 1 [42]. Sample sizes for each experiment are described in the figure legends. Plasma concentrations of 25-hydroxyvitamin D (vitamin D) were measured with electrochemiluminescent technology in a Cobas E411 Analyzer of Roche diagnostics (Indianapolis, IN, USA) at HCUCH.

### 2.3. VDR Genotyping

DNA was extracted from peripheral blood collected in EDTA-containing Vacutainer™ tubes using DNA Genomic PureLink^®^ Kit according to the manufacturer’s instructions. Following DNA extraction, all samples were quantified by absorbance using a Multiskan Go^®^ spectrophotometer of Thermo Fisher. DNA concentration and purity were assessed based on the 260/280 nm absorbance ratio (≥1.8). All samples were subsequently normalized to a standard concentration of 10 ng/µL, and DNA integrity was randomly verified by electrophoresis on a 1% agarose gel. The polymorphisms genotyping were analyzed by real-time PCR using TaqMan^®^ SNP Genotyping Assay according to the manufacturer’s instructions. The probes for VDR polymorphisms ApaI (rs7975232) and Taq (rs731236) were designed and synthesized by Applied Biosystems (Assay ID: C 28977635 10 for ApaI and C 2404008 10 for TaqI) [29,43].

### 2.4. Peripheral Blood Mononuclear Cell (PBMC) Isolation

Peripheral blood (15 mL) was obtained by venipuncture, and PBMCs were isolated by using the density gradient medium Lymphoprep and SepMate™ tubes. SepMate™ tubes were prepared by adding 15 mL of Lymphoprep at room temperature. Blood samples were diluted 1:1 with RPMI 1640 medium and carefully layered onto the SepMate™ tubes containing Lymphoprep. Tubes were centrifuged at 1200× *g* for 20 min at 20 °C, with acceleration and brake set to 0. The PBMCs layer was collected, washed with RPMI 1640 medium, and centrifuged at 600× *g* for 15 min at 20 °C in conic tubes. When necessary, residual erythrocytes were removed using 1× Ammonium–Chloride–Potassium (ACK) lysis buffer followed by centrifugation at 600× *g* for 7 min. Cells were resuspended in RPMI 1640 medium, and cell number and viability were determined by automated cell counting [44].

For cryopreservation, PBMCs were pelleted (600× *g*, 7 min), resuspended in cold CryoStor solution, and transferred to labeled cryovials. Vials were placed in a controlled-rate freezing container at −80 °C for at least 5 h before long-term storage in liquid nitrogen.

### 2.5. Viability Assays

One million PBMCs were cultured for 20 h at 37 °C in the presence of 20, 50 and 100 µM H_2_O_2_. Cells were harvested and stained with 10 mg/mL propidium iodide (PI) to determine cell viability by fluorescence-activated cell sorting (FACS) as described previously [15]. The fluorescence associated with total DNA content was defined by the permeabilization of cells with methanol. Samples were analyzed by flow cytometry in a FACScan (Becton Dickinson) using the software program FlowJo version 7.6.1 (Tree Star Inc., Ashland, OR, USA) [45].

### 2.6. Quantitative PCR

The mRNA levels were quantified by real-time PCR using Master Mix SsoAdvanced™ Universal SYBR^®^ Green Supermix according to the manufacturer’s instructions. All results were normalized to the geometric mean of 18S rRNA and SDHA housekeeping genes. The mRNA levels were calculated using the CT method. Table 2 shows the primer sequences for all genes.

### 2.7. Immunofluorescence

Immunofluorescence assays were conducted using 500,000 PBMCs. Cells were fixed with 1% paraformaldehyde for 10 min at room temperature, followed by PBS washes. Permeabilization was performed with 0.2% Triton X-100 for 5 min at room temperature, with subsequent PBS washes. Blocking was achieved using 2% BSA for 30 min at room temperature. The samples were then incubated overnight at 4 °C with a primary anti-Nrf2 antibody (1:1000 dilution in 0.2% BSA). After three PBS washes, cells were incubated with a secondary antibody (anti-mouse Alexa Fluor 488, 1:400 dilution in 0.2% BSA) for 1 h at room temperature. Nuclear counterstaining was performed using DAPI (1:10,000 dilution in PBS) for 5 min at room temperature. Finally, samples were mounted using Dako mounting medium. The images were acquired using a Nikon C2+ confocal microscope with a 60× objective. Image analysis was performed using Fiji (ImageJ version 1.54p).

### 2.8. Statistical Analysis

Differences between genotypes and comparisons of mRNA levels for each gene were assessed using the nonparametric Mann–Whitney test, as data did not follow a normal distribution according to the Shapiro–Wilk test (*n* < 50). In addition, a two-way ANOVA was performed to evaluate the effects of genotype and concentration, as well as their interaction. When significant effects were detected, multiple comparisons were performed using Bonferroni’s post hoc test. All statistical analyses were performed using GraphPad Prism 8 software (GraphPad Software, San Diego, CA, USA). Results are expressed as mean ± standard error of the mean (SEM).

## 3. Results

### 3.1. Demographic and Cognitive Characteristics of the Study Population

The study population consisted of cognitively healthy older adults, predominantly women (87%), with a mean age of 72.27 ± 6.4 years (Table 1). The participants had a mean educational level of 13.2 ± 3.2 years. Mean serum 25-hydroxyvitamin D (vitamin D) levels were 24.02 ± 9.1 ng/mL, with most participants presenting values within the range defined as vitamin D insufficiency (20–30 ng/mL). Regarding clinical characteristics, 63.3% of participants had a history of hypertension, 30% had type 2 diabetes mellitus, and 36.6% were current or former smokers. Cognitive performance was preserved across the cohort, with MoCA scores ranging from 25 to 30 (mean 28.14 ± 2) and MoCA-MIS values between 13 and 15 (mean 14.45 ± 0.8) (Table 1).

### 3.2. VDR Polymorphism Promotes a Differential Response to Oxidative Stress

We investigated the impact of the VDR ApaI (A > C) polymorphism on cellular susceptibility to oxidative stress-induced cell death. PBMCs viability of cognitively healthy older adults carrying the different alleles of the VDR ApaI (A > C) polymorphism was assessed after exposure to increasing concentrations of hydrogen peroxide (H_2_O_2_). The A allele has been described as protective, associated with a decreased risk of developing MCI, while the C allele has been associated with an increased risk of developing MCI [29]. To evaluate the influence of these polymorphisms, we assessed PBMCs viability under a dominant genetic model as previously we described [29], where the C allele exhibits dominance over the A allele [46]. Based on this, individuals were classified into two groups: homozygous carriers of the A allele (AA genotype) or as carriers of the C allele, either in heterozygous (AC) or homozygous (CC) form (AC/CC).

**Figure 1:**
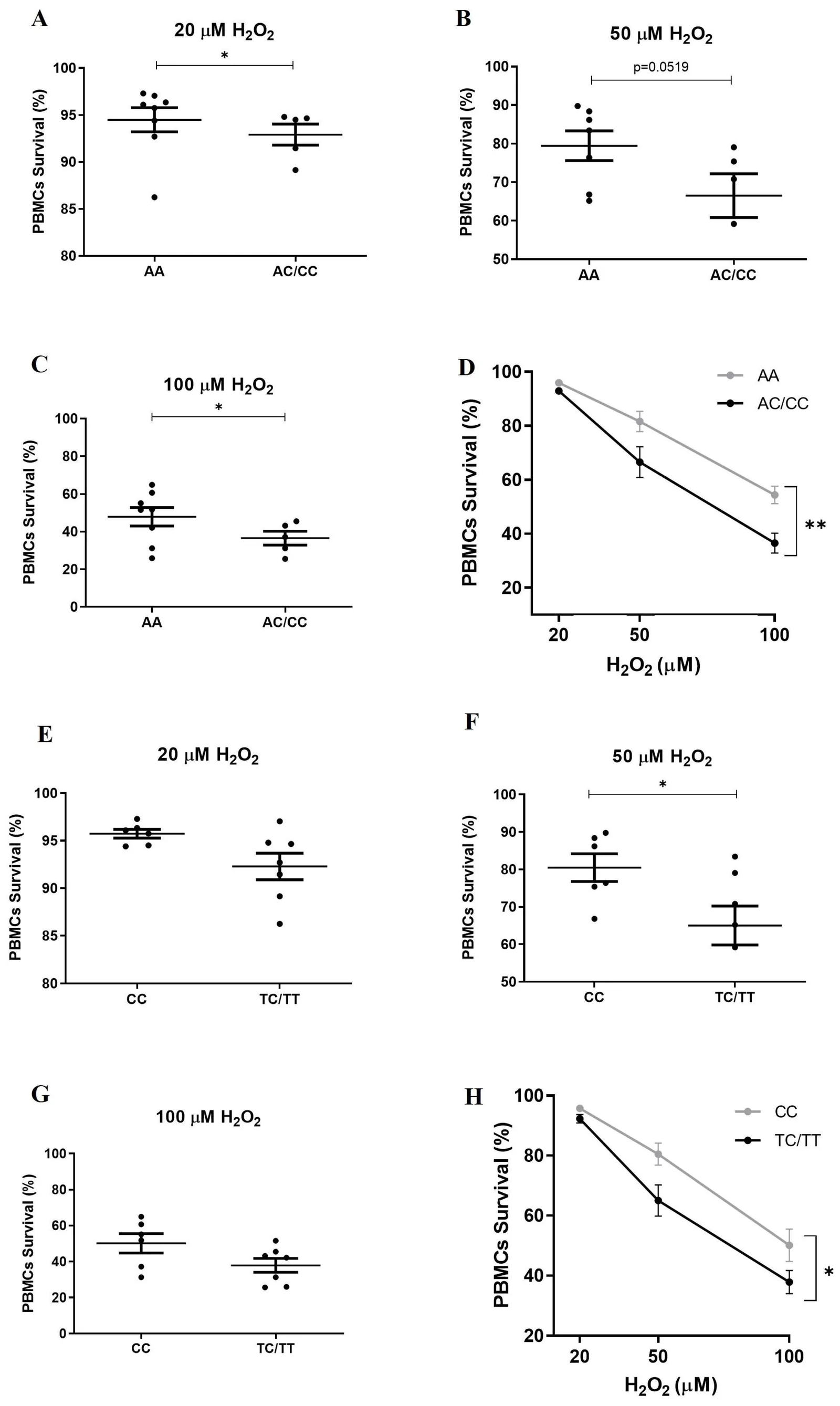
Differential oxidative stress responses are associated with VDR polymorphisms. PBMCs from cognitively healthy older adults were exposed to H_2_O_2_ (20, 50, or 100 µM) for 20 h, after which cell viability was assessed by flow cytometry. (**A**–**C**) H_2_O_2_-induced PBMC death according to ApaI genotype. (**D**) Analysis of concentration–genotype interaction. (**E**–**G**) H_2_O_2_-induced PBMC death according to TaqI genotype. (**H**) Analysis of concentration–genotype interaction. Statistical analysis: for panels (**A**–**C**,**E**–**G**), differences between genotypes were evaluated using Student’s *t*-test * *p* < 0.05. For panels (**D**,**H**), a two-way repeated-measures ANOVA followed by Bonferroni’s post hoc test was performed * *p* < 0.05; ** *p* < 0.005). For ApaI: AA *n =* 8; AC/CC *n* = 5. For TaqI: CC *n* = 6; TC/TT *n* = 7.

Cells were exposed to 20, 50 and 100 µM H_2_O_2_ for 20 h, after which cell viability was assessed by flow cytometry using Propidium Iodine (PI) staining. H_2_O_2_ induced a dose-dependent decrease in cell viability in both AA and AC/CC carriers. Notably, we found that cell viability was significantly higher in individuals with the AA genotype compared to those with the AC/CC genotypes at 20 µM H_2_O_2_, (Figure 1A) (AA 94.49 ± 3.6 vs. AC/CC 92.91 ± 2.52, *p* = 0.0303) and 100 µM H_2_O_2_ (Figure 1C) (AA 47.89 ± 13.77 vs. AC/CC 36.51 ± 8.27, *p* = 0.0397). Meanwhile, the difference at 50 µM H_2_O_2_ did not reach statistical significance, despite a *p* value close to the conventional threshold (AA 79.46 ± 10.17 vs. AC/CC 66.52 ± 12.70; *p* = 0.0519) (Figure 1B).

Two-way repeated-measures ANOVA (Figure 1D) revealed a significant effect of H_2_O_2_ concentration (*p* < 0.0001), a significant effect of genotype (*p* < 0.0176), and a significant interaction (concentration-genotype) (*p* < 0.0068). AA carriers consistently exhibited higher cell viability across increasing oxidative stress levels compared to AC/CC individuals.

We also analyzed the impact of the TaqI (C > T) polymorphism under a dominant model, where the C allele is protective and the T allele is considered a risk variant [29]. Individuals were classified into two groups: homozygous carriers of the protective C allele (CC genotype) and carriers of the risk T allele in heterozygous (TC) or homozygous (TT) form (TC/TT). PBMCs from CC individuals showed significantly higher viability than those from TC/TT carriers at 50 µM H_2_O_2_ (CC 80.49 ± 9.07 vs. CT/TT 65.01 ± 13.78, *p* = 0.0500) (Figure 1F). However, no significant differences were observed between the genotypes at 20 µM H_2_O_2_ (CC 95.73 ± 1.12 vs. CT/TT 92.29 ± 3.69, *p* = 0.1014) (Figure 1E) or 100 µM H_2_O_2_ (CC 50.13 ± 13.29 vs. CT/TT 37.85 ± 10.25, *p* = 0.0793) (Figure 1G).

Two-way repeated-measures ANOVA (Figure 1H) revealed a significant effect of H_2_O_2_ concentration (*p* < 0.0001), a significant effect of genotype (*p* < 0.0496), and a significant interaction (concentration-genotype) (*p* < 0.0465). CC carriers consistently exhibited higher cell viability across increasing oxidative stress levels compared to CT/TT individuals.

### 3.3. VDR Polymorphism Modulates the Expression of Nrf2 and Its Target Genes

Previously, we reported an association of the VDR polymorphism with mRNA levels of VDR and some of its target genes; for the ApaI polymorphism, the mRNA expression of VDR was higher in the AA genotype than in AC/CC in healthy older adults. In contrast, no differences in VDR mRNA expression were observed among TaqI genotypes [29].

Based on these results, we investigated the expression of Nrf2, a key transcription factor that regulates cellular redox balance and protective antioxidant responses. The presence of vitamin D response elements (VDREs) in the regulatory region of the Nrf2 gene suggests that its expression may differ depending on VDR polymorphisms. In addition, we also evaluated two Nrf2-regulated genes: heme oxygenase (HO) and Glutathione S-transferase (GST). HO, encoded by the *HMOX* gene, is an enzyme that catalyzes the degradation of heme into carbon monoxide, iron, and bilirubin, and the enzyme GST catalyzes the conjugation of toxic exogenous and endogenous compounds with reduced glutathione, facilitating detoxification.

**Figure 2:**
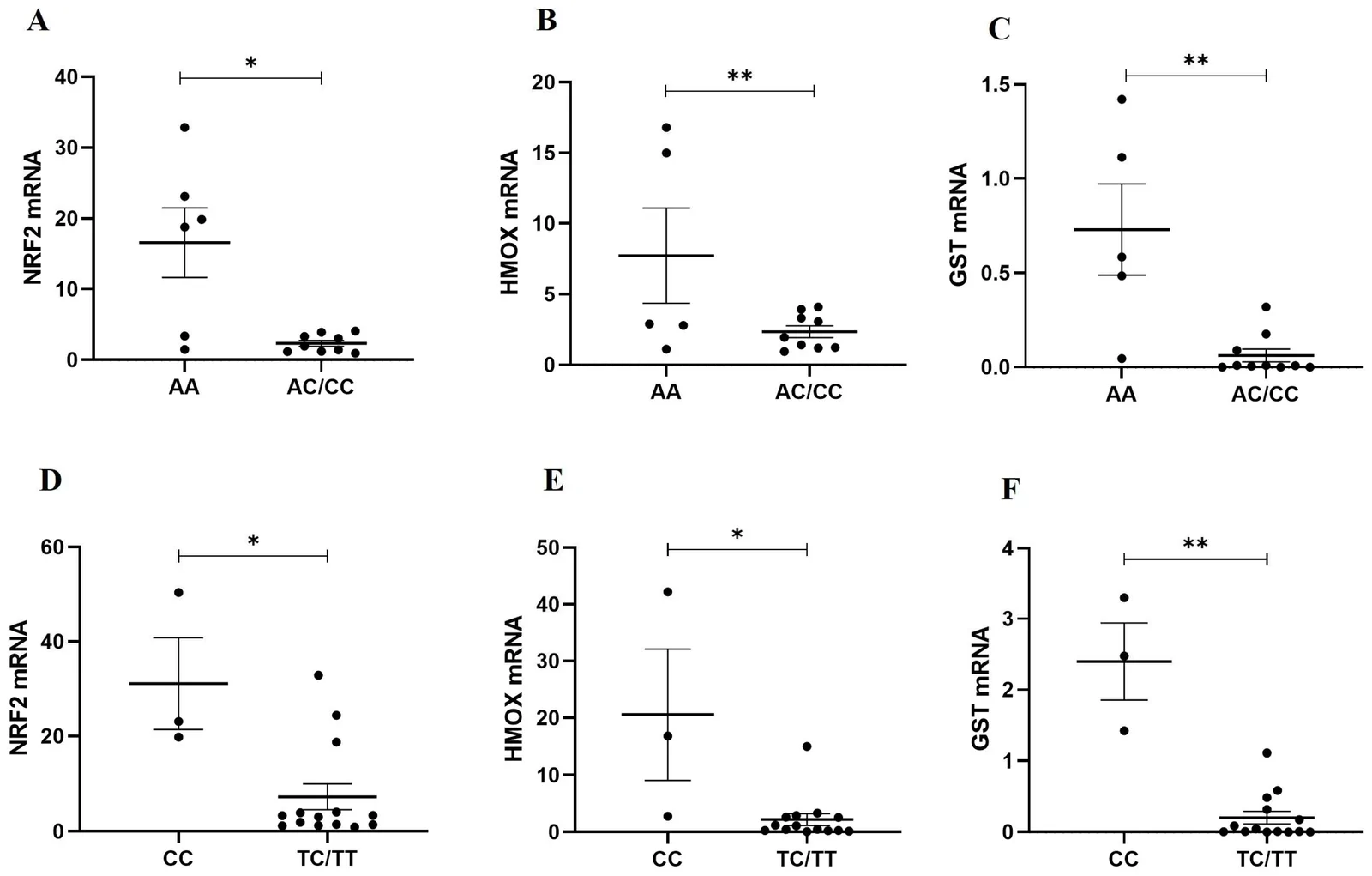
Nrf2 and Nrf2 target gene expressions are associated with VDR polymorphisms. mRNA expression levels of Nrf2, glutathione S-transferase (GST), and heme oxygenase-1 (HMOX1) were determined in PBMCs according to (**A**–**C**) Apa I and (**D**–**F**) Taq I genotypes by quantitative PCR (qPCR). Data were normalized to the housekeeping genes 18S and SDHA. Statistical analysis was performed using Student’s *t*-test. * *p* < 0.05; ** *p* < 0.005. For ApaI: AA *n* = 6; AC/CC, *n* = 9. For TaqI: CC *n* = 3; TC/TT *n* = 14.

For the ApaI polymorphism, the mRNA expression levels of Nrf2 (AA 16.57 ± 12.04 vs. AC/CC 2.34 ± 1.25, *p* = 0.0176), HMOX (AA 7.71 ± 7.52 vs. AC/CC 0.89 ± 1.01, *p* = 0.0070) and GTS (AA 0.73 ± 0.54 vs AC/TC 0.062 ± 0.11, *p* = 0.0047) were significantly higher in the AA genotype than in AC/CC (Figure 2A–C). In the same way, for TaqI polymorphism CC, individuals showed significantly higher mRNA level expression of Nrf2 (CC 31.11 ± 16.78 vs. CT/TT 7.29 ± 10.24, *p* = 0.0324), HMOX (CC 20.59 ± 19.97 vs. CT/TT 2.18 ± 3.86, *p* = 0.0206) and GTS (CC 2.40 ± 0.94 vs. CT/TT 0.20 ± 0.32, *p* = 0.0029) than those from CT/TT carriers (Figure 2D–F). These results support the hypothesis that the protective alleles, A for ApaI and C for TaqI, differentially regulate the antioxidant response of VDR target genes.

### 3.4. VDR ApaI–TaqI Haplotypes Are Associated with Differential Oxidative Stress Responses

Both ApaI and TaqI are located near the 3′UTR region of the VDR gene, and their effect on VDR function remains unknown. However, due to this proximity, ApaI and TaqI can be evaluated as associated haplotypes [44]. In this context, we considered the combination of protective alleles (A for ApaI and C for TaqI) as a protective haplotype and the risk alleles (C for ApaI and T for TaqI) as risk haplotypes.

**Figure 3:**
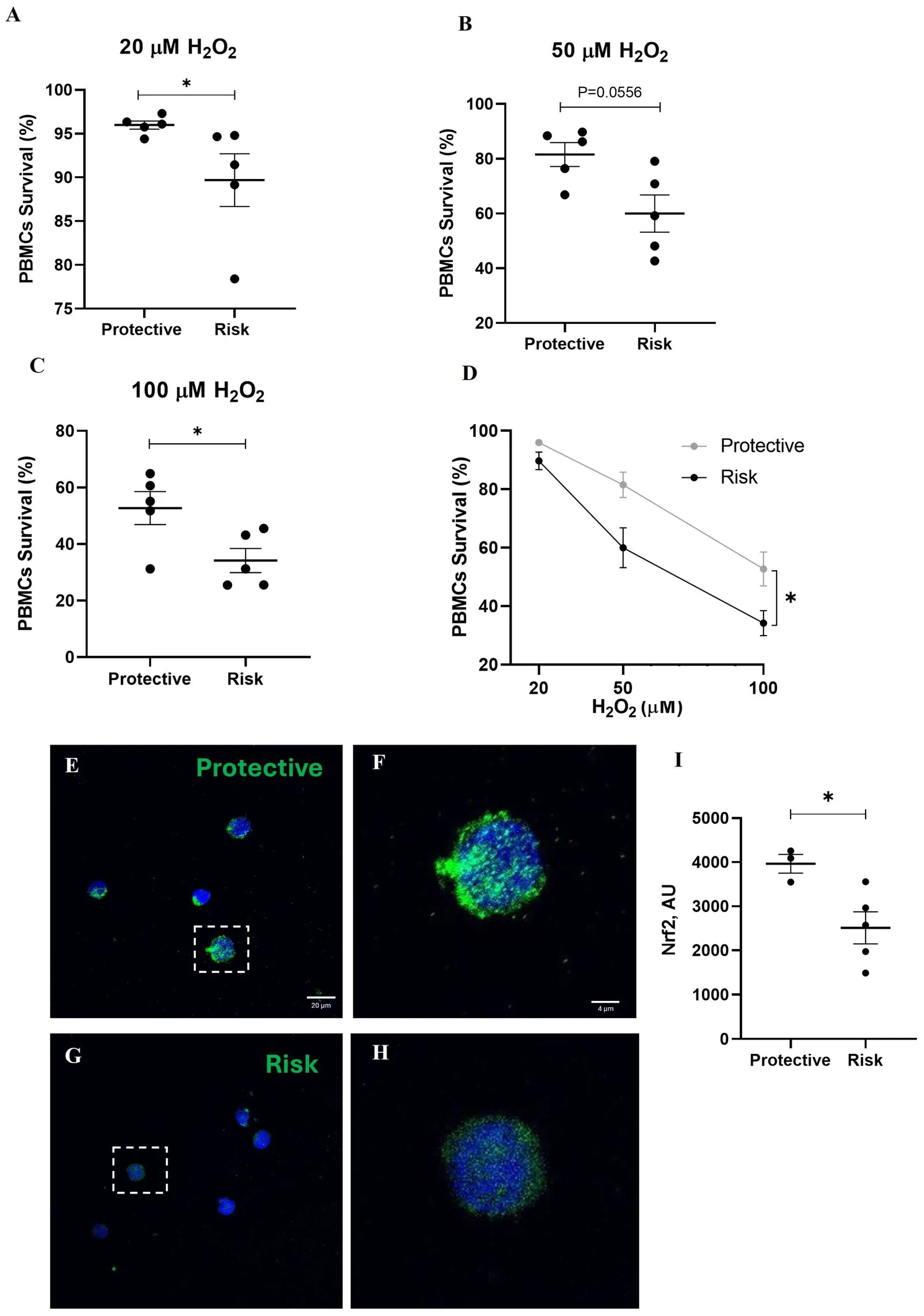
Differential oxidative stress responses according to VDR ApaI–TaqI haplotypes. ApaI and TaqI were evaluated as combined haplotypes. The protective haplotype carries the alleles A (ApaI) and C (TaqI), whereas the risk haplotype carries the alleles C (ApaI) and T (TaqI). (**A**) Percentage of PBMCs survival after 20 h exposed to 20 µM H_2_O_2_, (**B**) 50 µM H_2_O_2_ or (**C**) 100 µM H_2_O_2_. (**D**) Analysis of concentration–genotype interaction. (**E**) Representative image of Nrf2 immunofluorescence of PBMCs from carrier of protective haplotype and (**F**) magnification of the cell outlined with a dashed-line square. (**G**) Representative image from carrier of risk haplotype carries and (**H**) magnification of the cell outlined with a dashed-line square. (**I**) Image quantification in arbitrary units. Confocal images were captured with a Nikon C2 Plus microscope with a Plan-Apochromat 40× objective. Statistics analysis: for (**A**–**C**,**I**) panel *T*-test *: *p* < 0.05. For panels (**D**), a two-way repeated-measures ANOVA followed by Bonferroni’s post hoc test was performed * *p* < 0.05. Protective haplotypes *n* = 5; risk haplotypes *n* = 5.

When evaluating cell viability following H_2_O_2_ exposure, our results demonstrate that cell viability was significantly higher in the protective haplotype compared to the risk haplotype both at 20 µM (protective 95.98 ± 1.05 vs. risk 89.69 ± 6.74, *p* = 0.0317) (Figure 3A) and 100 µM H_2_O_2_ (protective 52.72 ± 13.05 vs. risk 34.18 ± 9.58; *p* = 0.0397) (Figure 3C). Additionally, a trend toward higher cell viability was observed in the protective haplotype at 50 µM H_2_O_2_ (protective: 81.51 ± 9.74 vs. 59.97 ± 15.18, *p* = 0.0556) (Figure 3B). Two-way repeated-measures ANOVA (Figure 3D) revealed a significant effect of H_2_O_2_ concentration (*p* < 0.0001), a significant effect of genotype (*p* < 0.0267), and a significant interaction (concentration-genotype) (*p* < 0.0286). Protective haplotype consistently exhibited higher cell viability across increasing oxidative stress levels compared to risk haplotype.

We also evaluated the protein expression of Nrf2 through immunofluorescence in PBMCs. Our results show that PBMCs from carriers of the protective haplotype exhibit significantly higher Nrf2 protein expression compared to those carriers of the risk haplotype (*p* = 0.0289) (Figure 3E–I).

## 4. Discussion

VDR signaling has emerged as an important modulator of aging-related processes, including oxidative stress, inflammation, and cellular survival. Growing evidence suggests that VDR can regulate transcription and activity of Nrf2, the master regulator of antioxidant response. However, the functional consequences of this pathway in the context of aging remain incompletely understood. In particular, the impact of genetic variability in VDR on the VDR–Nrf2 pathway has received limited attention.

We demonstrate that ApaI and TaqI VDR polymorphisms are associated with differences in susceptibility to oxidative stress-induced cell death in PBMCs from cognitively healthy older adults potentially mediated by differential regulation of oxidative stress response genes. Specifically, the protective alleles (A for ApaI and C for TaqI) were associated with increased cellular viability following H_2_O_2_ exposure compared with the risk alleles (C for ApaI and T for TaqI). Consistent with this phenotype, carriers of the protective alleles exhibited higher mRNA expression levels of Nrf2 and its downstream antioxidant genes HMOX and GTS than the risk alleles. Our results highlight the potential involvement of VDR genetic variability in particular ApaI and TaqI polymorphisms in cellular antioxidant responses through modulation of the VDR–Nrf2 signaling axis in cognitively healthy older adults.

Interestingly, although the ApaI and TaqI polymorphisms in the VDR gene are considered silent variants [47,48], the differential response observed across increasing H_2_O_2_ concentrations did not follow a strictly linear pattern, which may reflect the complex dynamics of redox adaptation rather than a simple dose-proportional effect. For the ApaI polymorphism, carriers of the protective allele in the homozygous state (AA) exhibited increased cell survival at both low (20 µM) and high (100 µM) H_2_O_2_ concentrations compared to carriers of the risk allele in homozygous and heterozygous states (AC/CC). Whereas at 50 µM, it did not reach statistical significance, the directionality of the effect remained consistent across concentrations, with AA carriers exhibiting higher viability at all tested doses.

In contrast, carriers of the protective allele for TaqI in the homozygous state (CC) showed increased cell survival compared with carriers of the risk allele (TC/TT) only at 50 µM H_2_O_2_. However, when the interaction between genotype and concentration was analyzed, the CC carriers exhibited higher PBMC survival across increasing oxidative stress levels compared to CT/TT individuals. The consistent directionality of the effect across all concentrations, together with the significant interaction term, supports a genotype-dependent modulation of oxidative stress responses associated with the ApaI and TaqI polymorphism.

These results suggest that the VDR polymorphism exhibited genotype-dependent differences in oxidative stress responses. Similarly, another study showed that the silent polymorphism in the multidrug resistance 1 (MDR1) gene has been shown to modify mRNA stability, leading to differences in expression and cellular responses to xenobiotic [49]. Consistently, silent polymorphisms in Catechol-O-methyltransferase (COMT) gene exhibited the largest differences in COMT enzymatic activity [50].

Also, we analyzed the expression of Nrf2 and its target genes, HMOX1 and GST, according to the protective and risk alleles of the ApaI and TaqI polymorphisms. Our results suggest that carriers of the protective alleles show higher mRNA levels of Nrf2 and its downstream targets compared with carriers of the risk alleles. Consistently, higher Nrf2 protein levels were also observed in individuals with the protective alleles, whereas the risk alleles are associated with a reduced antioxidant response capacity. These results support the hypothesis that protective alleles differentially regulate susceptibility to oxidative stress-induced cell death in cognitively healthy older adults, likely through the antioxidant response of VDR-Nrf2 target genes. Although these results are relevant, they should be interpreted with caution. The ApaI and TaqI polymorphisms of the VDR gene are considered silent variants [48], and polymorphisms located in the 3′ region may influence transcript stability or be in linkage disequilibrium with other functional variants. Moreover, mRNA levels do not always reflect protein abundance due to post-transcriptional regulatory mechanisms. Therefore, further studies will be required to confirm these findings at the protein level in HMOX1 and GST.

The link between VDR polymorphisms and antioxidant capacity has received limited attention. In children, the allele A of ApaI is associated with markers of inflammation and oxidative stress, particularly diminished levels of serum TNF-α and 8-iso-PGF2α. Moreover, the combination of specific haplotypes of FokI, ApaI and TaqI—ACA, AAA, GAA—was associated with high levels of HDL, low levels of TNF-α and 8-iso-PGF2α, indicating that VDR polymorphisms may influence susceptibility to, or protection against, the development of cardiovascular diseases [51]. Also associated with cardiovascular diseases, it has been reported that individuals homozygous for the A allele of ApaI show the lowest means for systolic and diastolic blood pressure, and homozygous CC had the highest values for blood pressure, indicating that the A allele was protective [46].

Nrf2 is a transcription factor recognized as a key regulator of the antioxidant response. Its expression has been shown to decline with age. Disruption of Nrf2 signaling is associated with an increased susceptibility to oxidative insults and other toxicants in humans and model organisms [52,53]. Accordingly, Nrf2 signaling is involved in longevity-associated pathways, contributing to extended lifespan and improved health span in long-lived species and humans [54]. Considering all this background, we demonstrate that variations in the VDR gene influence Nrf2 levels and the expression of its target genes, thereby accounting for the differential susceptibility to oxidative stress-induced cell death. Thus, cells from individuals of the same age exhibit different responses to oxidative stress, highlighting a genetically influenced response that may act as a potential oxidative biomarker for determining an individual’s oxidative stress resilience. We propose the concept of oxidative stress resilience as the capacity of cells to preserve viability and functional homeostasis under excessive oxidative stress exposure through coordinated activation of antioxidant defenses and cytoprotective mechanisms [55,56]. The enhancement of oxidative stress resilience facilitates the clearance of excess oxidants and mitigates the risk of severe oxidative injury. From this perspective, the A allele of ApaI and the C allele of TaqI represent VDR variants that confer oxidative stress resilience, promoting healthier aging. Moreover, given the proximity between the ApaI and TaqI polymorphisms, these variants can be evaluated as associated haplotypes [47]. We demonstrated that protective haplotypes are associated with higher mRNA expression levels of antioxidant response genes compared with risk haplotypes. This haplotype may contribute to increased resilience to oxidative stress-associated diseases in older adults.

Numerous studies have demonstrated that age-related oxidative damage is characterized by increased oxidant production and diminished antioxidant defenses, alterations that are further exacerbated in individuals with MCI and AD patients [57,58,59,60]. Increased oxidative stress and impaired protein homeostasis have been consistently reported in both central and peripheral tissues of individuals with MCI and AD, suggesting that redox dysregulation is a hallmark of neurodegenerative vulnerability [61,62,63,64,65]. Meanwhile, genetic variability can produce alternative alleles that may serve as either protective factors or risk factors for diseases [56,65]. This variability has enabled the development of scoring tools to assess resilience. We propose that oxidative stress resilience is influenced by genetic variation and that this variability may contribute to either protection against or acceleration of neurodegenerative diseases. Future evaluation in prospective cohorts is warranted to examine MCI incidence and AD progression among carriers of protective or risk VDR alleles and to determine whether vitamin D status modifies these genotype-dependent effects.

The limitations of this study are substantial, yet it provides a valuable starting point for future research. Key limitations include the small number of participants and that the cohort includes a high proportion of women. Historically, biomedical and clinical research has exhibited sex bias [66], with studies enrolling significantly more male than female participants in many fields, which has negatively impacted the understanding, detection, and treatment of disease in women. The unequal representation of both sexes, combined with the small number of participants, constitutes a significant limitation, positioning this study as a pilot effort that provides a basis for future, larger-scale investigations. Another limitation is the assessment of genetic variability in a single gene and only two of its polymorphisms. These variants were selected based on prior evidence of their relevance in diseases linked to unhealthy aging, including AD, diabetes mellitus, and other metabolic and age-associated conditions. Despite these constraints, the positive findings support the rationale for expanding the number of genes and variants evaluated in future prospective cohorts with larger sample sizes, to further validate and broaden our hypothesis.

## 5. Conclusions

Our findings indicate that VDR polymorphisms are associated with differential Nrf2-dependent oxidative stress responses, suggesting that genetic variation in the VDR gene may modulate cellular resilience to oxidative challenges. This variability may contribute to differences in the ability of cognitively healthy older adults to cope with oxidative stress. Understanding the genetic determinants of antioxidant responses could support the development of personalized strategies aimed at promoting healthy aging and longevity. Moreover, vitamin D status or supplementation may differentially influence oxidative stress responses according to VDR genotype.

## Figures and Tables

**Table 1:** Demographics and Clinical Characterization.

Variable	Total Cohort (*n* = 30)
Age (years), mean ± SD	72.27 ± 6.4
Sex, woman (%)	86.7%
Education (years), mean ± SD	13.20 ± 3.2
25(OH)D (ng/mL), mean ± SD	24.02 ± 9.1
MOCA	28.10 ± 2.0
MOCA-MIS	14.47 ± 0.8
Hypertension, *n* (%)	63.3%
Diabetes mellitus, *n* (%)	30%
Smoking status, *n* (%)	36.6%

**Table 2:** Primer sequences.

Name	Accession Number	Primer Forward	Primer Reverse
**Nrf2**	NM_006164	ATGGATTTGATTGACATACTT	ACTGAGCCTGATTAGTAGCAAT
**HMOX1**	NM_002133.3	ATGCCCCAGGATTTGTCAGA	AAGTAGACAGGGGCGAAGAC
**GSTT1**	NM_000853	TGCCGCGCTGTTTACATCTT	GTGCTGACCTTTAATCAGATCCA
**18S**	NM 022551	GCGAGTACTCAACACCAACACCAACATC	CCTCAACACCACATGAGCATA TC
**SDHA**	NM_004168	GAGGCAGGGTTTAATACAGCA	CCAGTTGTCCTCCTCCATGT

## Data Availability

The original contributions presented in this study are included in the article. Further inquiries can be directed to the corresponding author.

## Abbreviations

The following abbreviations are used in this manuscript:

AD	Alzheimer disease
GST	Glutathione S-transferase
HO	heme oxygenase
HMOX1	heme oxygenase-1
MCI	Mild Cognitive Impairment
MOCA	Montreal Cognitive Assessment
NRF2	Nuclear factor erythroid 2-related factor 2
PBMC	Peripheral blood mononuclear cells
PCR	Polymerase chain reaction
ROS	Reactive species of oxygen
RXR	Retinoid X Receptor
SNP	Single-nucleotide polymorphisms
VDR	Vitamin D receptor
